# Analysis of imaging signatures in ^18^F-DOPA PET of glioblastoma treated with dose-escalated radiotherapy

**DOI:** 10.3389/fonc.2025.1623313

**Published:** 2025-08-13

**Authors:** Jing Qian, Deanna H. Pafundi, William G. Breen, Paul D. Brown, Christopher H. Hunt, Mark S. Jacobson, Derek R. Johnson, Timothy J. Kaufmann, Bradley J. Kemp, Sani H. Kizilbash, Val J. Lowe, Michael W. Ruff, Jann N. Sarkaria, Joon H. Uhm, Mark J. Zakhary, Maasa H. Seaberg, Hok Seum Wan Chan Tseung, Elizabeth S. Yan, Yan Zhang, Nadia N. Laack, Debra H. Brinkmann

**Affiliations:** ^1^ Department of Radiation Oncology, Mayo Clinic, Rochester, MN, United States; ^2^ Department of Radiation Oncology, Mayo Clinic, Jacksonville, FL, United States; ^3^ Department of Radiology, Mayo Clinic, Rochester, MN, United States; ^4^ Department of Medical Oncology, Mayo Clinic, Rochester, MN, United States; ^5^ Department of Neurology, Mayo Clinic, Rochester, MN, United States; ^6^ Department of Radiation Oncology, University of Maryland, Baltimore, MD, United States; ^7^ Department of Radiation Oncology, University of California San Francisco Medical Center, San Francisco, CA, United States

**Keywords:** glioblastoma, radiotherapy, 18 F-DOPA PET, treatment response, quantitative imaging

## Abstract

**Background/objectives:**

^18^F-DOPA is an amino acid radiotracer with high uptake in glioblastoma and low uptake in normal brain. Patients underwent pre-radiation and post-radiation ^18^F-DOPA PET scans on a prospective clinical trial. This analysis investigates quantitative image features correlated with prognosis and treatment response to identify patients who benefit the most from dose-escalated therapy.

**Methods:**

Quantitative image features from ^18^F-DOPA PET scans of 58 glioblastoma patients were extracted from the high uptake region (TBR>2.0) in both pre-RT and early post-RT follow-up PET images, which were then refined using Pearson pair correlation. To explore the possibility to identify patients who benefit the most from dose-escalated therapy, pre-irradiation features were identified with univariate Cox regression analysis. Classifications with simple threshold or with Decision Tree models were carried out to categorize patients into distinct survival groups. Additionally, the features with notable changes before and after RT were identified and the temporal patterns of these changes between the survival groups were compared. Multivariates cox analysis was performed to assess the prognostic value of delta features in survival analysis.

**Results:**

The pre-irradiation features demonstrated predictive capability in distinguishing survival groups, yielding an accuracy of 0.78 on the reserved test dataset. We also pinpointed eight quantitative features that exhibited a significant difference before and after radiotherapy in patients with MGMT-unmethylated glioblastoma. The change of the features presented different patterns between the survival groups separated by median overall survival and the inclusion of delta features can enhance the accuracy of survival analysis. Conversely, for patients with methylated MGMT, no feature displayed such significant changes between preRT and early postRT.

**Conclusions:**

Our study showcased the potential of employing quantitative features derived from ^18^F-DOPA images to refine the stratification of patients with unmethylated MGMT for dose escalated therapy. Moreover, the change of these features can serve as valuable tools for monitoring treatment responses following radiotherapy.

## Introduction

Glioblastoma (1) is typically treated with maximally safe surgical resection followed by radiotherapy and concomitant and adjuvant chemotherapy, with or without tumor treating fields (2, 3). Despite these incremental advances in multimodality treatment, survival for many patients remains poor with a median survival of 15 months after diagnosis (4). The efficacy of treatment also showed strong correlation with certain biomarkers, for example, patients with methylated O^6^-methylguanine methyltransferase (MGMT) promoter often benefit from temozolomide while patients with unmethylated MGMT do not (5), although the heterogeneity of glioblastoma and variability in MGMT expression across tumor regions complicates the correlation between MGMT expression and treatment response (6, 7). The IDH (isocitrate dehydrogenase) status is another important molecular characteristic and prognostic indicator in glioblastoma. Mutant IDH is generally associated with a better prognosis and longer survival than IDH-wildtype glioblastoma (6, 8). Magnetic resonance imaging (MRI) including T1 post-gadolinium and T2 series are the standard for diagnosis, treatment planning and follow-up of glioblastoma, however as the images providing only morphological information, their sensitivity and capability to distinguish tumor from treatment effects are limited (9, 10). Improvements in assessment of treatment response and tumor progression may result from advances in imaging with modalities such as advanced MRI (i.e. perfusion and diffusion MRI (4, 11–14) or PET with amino acid tracers (15–19). As one of the most promising techniques, amino acid PET images provide more specific uptake in tumor tissue than in areas of radiation-induced normal tissue response (20, 21) and have been recommended for assessing glioma progression (22, 23).

Quantitative analysis of amino-acid PET imaging is of significant interest in glioblastoma diagnosis (24, 25) and monitoring (26, 27) due to its ability to quantitatively capture tumor heterogeneity and other prognostic information (28, 29). This analysis could utilize radiomics or other mathematical tools to extract quantitative image features. Traditional radiomics studies (24–27, 30) typically focus on single-time-point data from patients treated with a standard protocol (2). Although it is essential to provide prognostic prediction before the start of therapy, information at a single time point could be restrained in assessing treatment responses to interventions such as radiotherapy (RT) while understanding treatment response is crucial for the post-therapy management. Additionally, although biomarkers like MGMT methylation are strong prognostic indicators of survival (5), they are often excluded in radiomics analysis possibly due to limited data availability. Given the varying survival rates of glioblastoma patients, exploring the potential of quantitative imaging to further stratify patients with the same biomarkers for individualized treatment and post-treatment management is of great interest.

To further improve treatment efficacy, clinical trials suggest that radiation dose escalation targeted to tumor heterogeneity may enhance patient survival (31, 32). However, not all patients benefit from increased radiation doses, and higher doses inevitably raise normal tissue toxicity and complicate post-therapy management. Therefore, identifying patients who may benefit from dose escalation is essential for personalized treatment. To address this challenge, in this study, we extracted quantitative image features from ^18^F-DOPA PET images of newly diagnosed glioblastoma patients undergoing dose-escalated RT (DERT), at pre-RT and serial post-RT follow-up (FU) timepoints, then performed single-time-point and time-series analyses on the extracted quantitative features. We aimed to identify pre-irradiation radiomic features to further stratify patients with the same MGMT methylation status, in order to determine which patients may benefit most from DERT for more individualized therapy. Additionally, we examined changes in the quantitative features associated with overall survival (OS), highlighting the survival-related response following DECT. Our study is the first to stratify glioblastoma patients for dose escalation, demonstrating the prognostic value of ^18^F-DOPA and its potential role in monitoring treatment response.

## Materials and methods

### Patient cohort

This study included patients with newly diagnosed glioblastoma treated with ^18^F-DOPA guided DERT (31) on an institutional prospective phase II clinical trial (NCT01991977). Patients enrolled in the trial were treated with chemoradiation with a boost to 76 Gy in 30 fractions guided by ^18^F-DOPA PET imaging, followed by standard adjuvant temozolomide. Surgical resection took place prior to the acquisition of any images investigated in this study. Extent of resection was categorized as biopsy, subtotal resection or complete resection. MGMT methylation status was defined using a clinical, quantitative methylation-specific PCR assay. The current study is a retrospective analysis of data curated from that clinical trial (31). To simplify the impact from different pathological biomarkers and because most patients in the trial had wild-type IDH, the patients with mutant IDH status were excluded in this study. All the patients in the trial with a pre-RT and at least one FU ^18^F-DOPA PET/CT scan were included in this current study. The first FU (FU1) images were acquired consistently at 1 month after completing RT with a mean of 32 days from the last session of RT and a standard deviation of 6.5 days, and subsequent FU frequency was determined for each patient based on clinical judgement and availability. The second FU (FU2) has a mean of 82 days from the last session of RT with a standard deviation of 18 days. The distribution of FU1 and FU2 timeframes can be found in **Supplementary Figure S1**. In this study, only the images at pre-RT, FU1, and FU2 timepoints were considered. Patients were first grouped based on their MGMT methylation status. Within each methylation status grouping, median OS was used as a threshold to classify patients into two subgroups: those with longer survival (LS), defined as OS above the median, and those with shorter survival (SS), defined as OS at or below the median. Informed consent was obtained from all patients, and the study was approved by Institutional Review Board (IRB) and complied with the principles of the Helsinki declaration.

### PET imaging and radiomics feature extraction

PET imaging was conducted on either a GE Discovery 690XT or a GE Discovery MI PET/CT system with matched spatial resolution, following a strictly controlled protocol (31, 33). ^18^F-DOPA was injected intravenously at a dose of 5 mCi ± 10%. PET sinograms were reconstructed using a fully 3-dimensional iterative reconstruction algorithm with corrections for attenuation, scatter, randomness, deadtime, decay, and normalization applied. All PET images were resampled into voxel dimensions of 2x2x2 mm. PyRadiomics (34) was employed to extract 26 shape, 19 first-order and 70 texture quantitative features from each scan, adhering to the definitions outlined by the Image Biomarker Standardization Initiative (35, 36). The list of extracted features is reported in **Supplementary Table S1**. All shape features are reported in voxel-based units. Additionally, relative delta features were calculated, representing the percentage change of a quantitative feature at a given FU timepoint compared to the pre-RT measurement for each patient.


$$\Delta Feature\left(t:FU\right)=100*\frac{Feature\left(t:FU\right)-Feature\left(t:pre-RT\right)}{Feature\left(t:pre-RT\right)}$$

All features were extracted from the region of high SUV uptake, where the tumor-to-normal-brain-tissue SUV ratio (TBR) exceeded 2.0. The SUVmean of the normal brain tissue was calculated on a wedge of the contralateral brain (33). This high uptake region, referred to as the region of interest (ROI) hereafter, corresponded to the region of dense tumor and exhibited greater predictive value compared to the entire tumor volume, as demonstrated in our prior work (33). The use of an SUV-threshold-based autosegmentation method also eliminated observer bias during tumor delineation. ROIs in each scan were reviewed by an experienced medical physicist and an experienced nuclear medicine physician to exclude physiological uptake, for example striatal uptake.

### Feature selection

To explore single timepoint classification into LS and SS survival groups based on pre-RT images, only shape and first-order features were utilized due to their straight-forward interpretation and ease of generalization in future work. For transparency, an exploratory Random Forest model including feature importance analysis is provided in the **Supplementary Material**. Although texture features may improve model performance, restriction to shape and first order features may improve reliability for datasets with limited size. These features were first filtered with a pairwise correlation coefficient below 0.8, followed by a univariate Cox regression analysis against OS. Only the features with hazard ratio (HR) out of the range [0.99, 1.01] and the p-value less than 0.05 were chosen.

To investigate features correlated to survival-associated treatment response, the feature selection principle involves utilizing features that undergo the most significant changes before and after RT. Therefore, delta quantitative features were employed on an individual variable basis. All extracted features, irrespective of their predictive nature, were incorporated into the analysis. Feature selection was guided by the magnitude of difference observed between the two OS groups, categorized by median OS as the threshold. Specifically, features were chosen if both mean and median values demonstrated a difference of more than 50% between the two survival groups for either FU1 or FU2, and the p-value of the feature was smaller than 0.05 for the LS versus SS comparison. Although all selected features underwent investigation, only those with a pairwise correlation coefficient below 0.8 are presented here to avoid redundancy.

### Classification model on pre-RT images

To assess the predictive capability of quantitative features to further stratify patients with the same biomarkers, we conducted simple threshold-based classification with each identified feature and also constructed classification models. Aiming to identify patients who may benefit from DERT, the classification model only utilizes the pre-RT images which were obtained before the DERT started. For each MGMT methylation status, the patient cohort with were split randomly with 75:25 ratio into train and test dataset. Given the modest size of the patient cohort in this study, considerations for simplicity, interpretability and reproducibility primarily dictated the choices of features and model algorithms. For each identified feature, a simple threshold search was performed on the train dataset to find out the cutoff for best accuracy, and that threshold was applied to the reserved test dataset. For a more complicated model, we opted to use the highly interpretable Decision Tree (DT) Classifier (37), implemented in Scikit-Learn python package (version 1.3.1) (38). The maximum depth of the DT and the maximum number of leaf nodes were both set to 2. The input features were restricted to those identified with univariate Cox analysis, with no attempt made to employ additional features or more complex algorithms to avoid overfitting concerns. Five-fold cross-validation was applied in the model fitting on the train dataset. The prediction on the test data is the average of the predictions from the trained five-fold classification models.

### Survival analysis with early FU images

After therapy, it becomes crucial to estimate how long a patient might survive at each FU timepoint. This is expressed as remaining survival (RS), defined as the time between a FU timepoint and the patient’s death. Understanding RS enables timely interventions for post-treatment management. RS reflects a combination of factors, including pre-treatment disease characteristics, treatment response, and tumor progression. For each patient, RS was calculated at each FU timepoint and used as the survivals in our analysis. Using early FU imaging, we conducted univariate and multivariate Cox regression analysis to fit RS. To ensure simplicity and reproducibility, we focused exclusively on the identified features in the shape and first-order categories for this analysis. The number of variables was limited to three to minimize the risk of overfitting. By analyzing changes in the Concordance index (C-index) with the inclusion of static and delta radiomic features at corresponding FU timepoints, we evaluated the prognostic value of delta features in predicting RS for patients in this cohort.

## Results

### Patient

**Table 1** displays the categorization of the IDH wild-type patients based on their MGMT status and median OS. Additional clinical details are available in the previous publication of the clinical trial (31). Among patients with unmethylated MGMT, the OS spanned from 5 to 41 months, with a median of 15 months. In contrast, patients with methylated MGMT exhibited an OS ranging from 17 months to over 74 months, with a median of 38 months. The distribution of age and gender is detailed in **Table 1**, revealing no discernible differences between the various survival groups. The distribution of re-section extent for each subgroup and the p-value of the group comparison is reported in **Table 1**. The p-values for the age, gender distributions and resection extent between SS and LS group are calculated with Mann-Whitney test.

**Table 1:** Epidemiology of the selected patients, categorized by MGMT methylation status and median OS.

MGMT methylation	Methylated		Unmethylated	
**Median OS [months]**	**38**		15	
Survival Group	SS	LS	SS	LS
Patient count	12	11	19	16
Male: Female	50%:50%	64%:36%	58%:42%	56%:44%
p-value: gender	0.543		0.422	
Age [year]: Min: Max	42:68	39:74	42:77	19:70
Age [year]: Median	59	59	59	55
p-value: age	0.828		0.184	
Resection: biopsy:subtotal:total	11%: 58%:32%	25%:31%:44%	8%:42%:50%	10%:45%:45%
p-value: resection	0.97		0.86	

### Feature selection

With univariate Cox regression analysis, only two pre-RT features were identified with strong correlation with OS for the patients with unmethylated MGMT status. Their hazard ratios, confidence interval (CI) and p-values are summarized in **Table 2**. Maximum is the maximum value of Tumor to Brain Ratio (TBRmax) reported in other literature (39–41). Skewness measures the asymmetry of the distribution of values about the mean TBR value. The same analysis was also applied to the cohort with methylated MGMT status, but no feature satisfying the criteria was identified.

**Table 2:** Summary of univariate Cox regression analysis for pre-RT single timepoint features that have strong correlation with OS for IDH wild-type patients with unmethylated MGMT status.

Feature	HR	95% CI lower	95% CI upper	p-value
Maximum (TBRmax)	1.33	1.07	1.66	0.01
Skewness	3.45	1.80	6.60	<0.01

**Table 3** presents the delta features that exhibited a significant difference before and after RT as well as between LS and SS groups. Among patients with unmethylated MGMT, eight such features were identified, whereas no delta feature was found to exhibit a significant difference at the same level (more than 50% for both mean and median) for patients with methylated MGMT. For a concise reference, a simplified description of these features is provided in **Supplementary Table S2**, although a detailed mathematical definition can be found in the PyRadiomics reference (34).

**Table 3:** Delta features which showed a significant difference between pre-RT images and FU1 or FU2 images.

Delta feature number	Category	Features	FU1 p-value	FU2 p-value
Unmethylated MGMT				
DF1	Shape	ΔMeshVolume	**0.038**	0.323
DF2		ΔSurfaceVolumeRatio	0.528	**0.012**
DF3	First order	ΔEnergy	**0.045**	0.303
DF4	Texture	gldm_ΔGrayLevelNonUniformity	**0.029**	0.265
DF5		glrlm_ΔShortRunHighGrayLevelEmphasis	0.417	**0.007**
DF6		glcm_ΔContrast	0.928	**0.027**
DF7		glszm_ΔSmallAreaHighGrayLevelEmphasis	**0.016**	0.044
DF8		glszm_ΔZonePercentage	0.601	**0.014**

The first column is assigned feature number, the second column and the third column show the feature category and name. The last two columns show the p-value of the feature between LS and SS comparison, in FU1 and FU2 image respectively.

P-value less than 0.05 are labeled in bold.

### Classification modeling on pre-RT images

Based on the train dataset, the optimized threshold of TBRmax was determined to be between [2.9, 3.2] for the MGMT unmethylated cohort, which gave an accuracy of 0.73 on the train dataset for LS/SS classification and 0.78 on the reserved test dataset. The F1 score for TBRmax was 0.67 on the test dataset. The optimized cutoff value of skewness was determined to be between [0.65, 0.75] which gave an accuracy of 0.73 on the train dataset and 0.67 on the test dataset. The F1 score for skewness was 0.73 on the test dataset. As a contrast, neither TBRmax nor skewness showed prognostic value for the MGMT methylated cohort, with best accuracy achieved of only 0.56.

**Figure 1:**
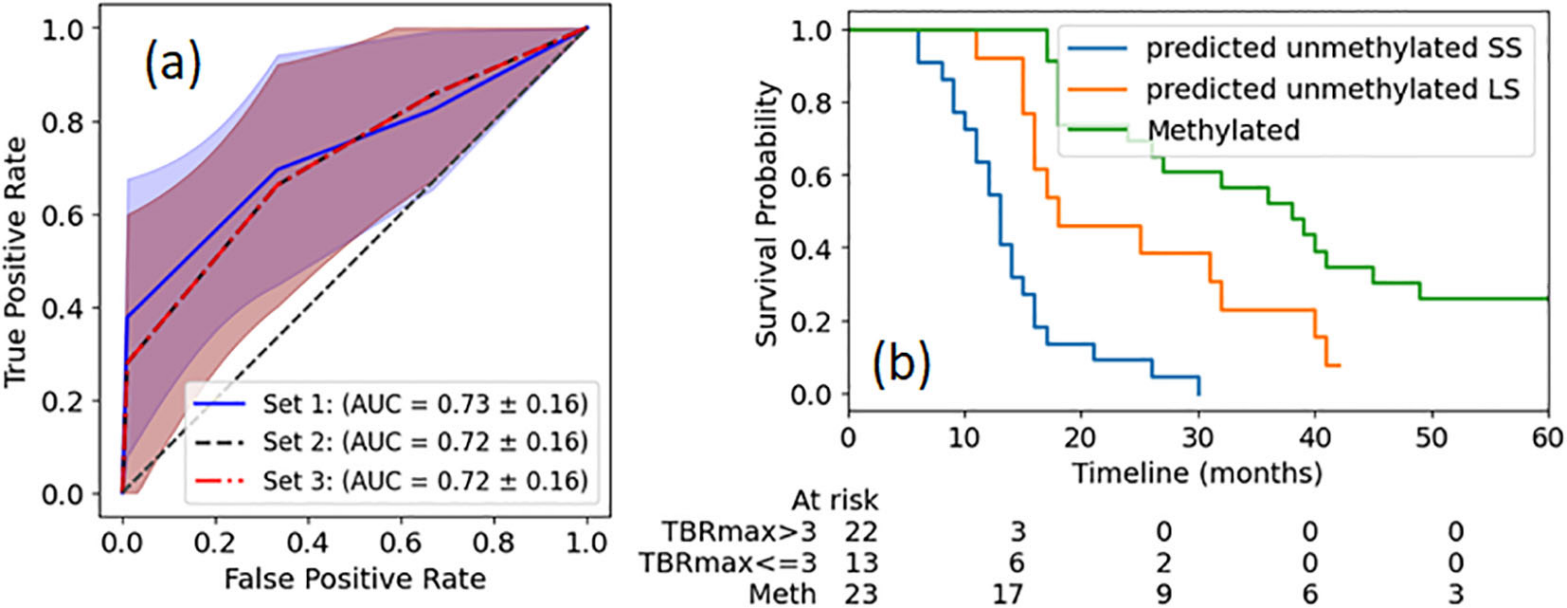
ROI (magenta contour) where the TBR ratio exceeded 2.0 for three example patients with different OS at the time points of preRT, FU1 and FU2. The patients with worse outcomes usually have increased SurfaceVolumeRatio.

To evaluate whether prognostic value could be improved with a more complex model, decision tree models were constructed using three different feature sets: Set 1 (TBRmax), Set 2 (Skewness) and Set 3 (TBRmax and Skewness). Training of the model with 5-fold cross validation on the train dataset (**Figure 1a**) demonstrated that the model with TBRmax still shows slightly better performance than models with other sets, although the difference is not significant. When applying the DT model with TBRmax to the reserved test dataset, an accuracy of 0.78 and a F1 score of 0.67 was obtained, showing no improvement compared to the simple cutoff method. With TBRmax = 3.0 as a cutoff, patients with unmethylated MGMT are grouped into SS and LS survival categories, with their Kaplan-Meier (KM) plots shown in **Figure 1b**. Additionally, the KM curve for patients with methylated MGMT is provided for reference.

### Time series of quantitative features

**Figure 2:**
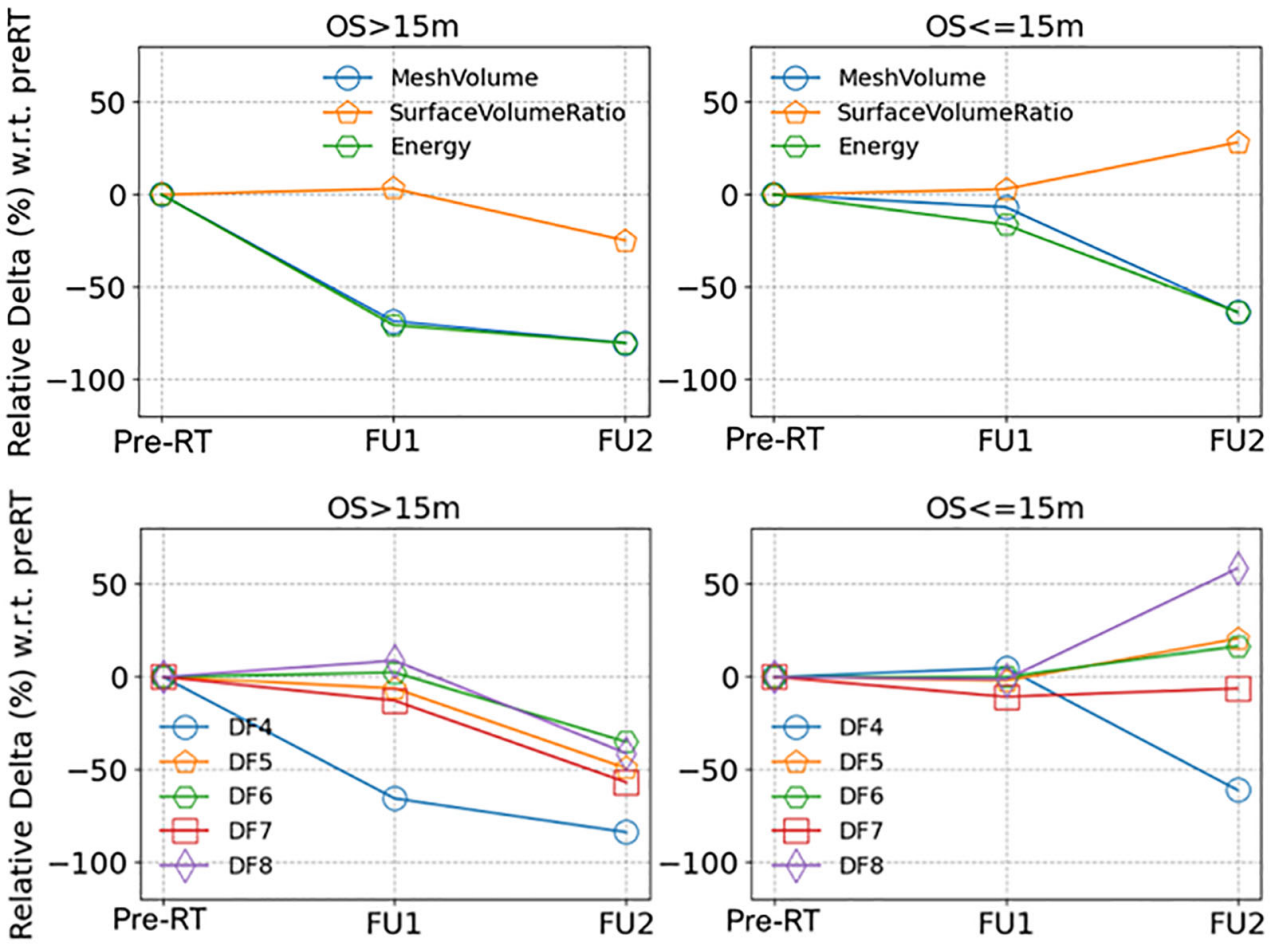
**(a)** ROC curves for survival group classification of MGMT unmethylated patients based on identified radiomics features extracted from single timepoint pre-RT ^18^F-DOPA PET images. The definitions of feature sets are described in the text. The shadow region depicts the 1 standard deviation. **(b)** KM plots of OS of patients with unmethylated MGMT status, separated by TBRmax cutoff. For reference, KM plot of OS of MGMT methylated patients is also plotted. The p value between any two groups is less than 0.01.

**Figure 3:**
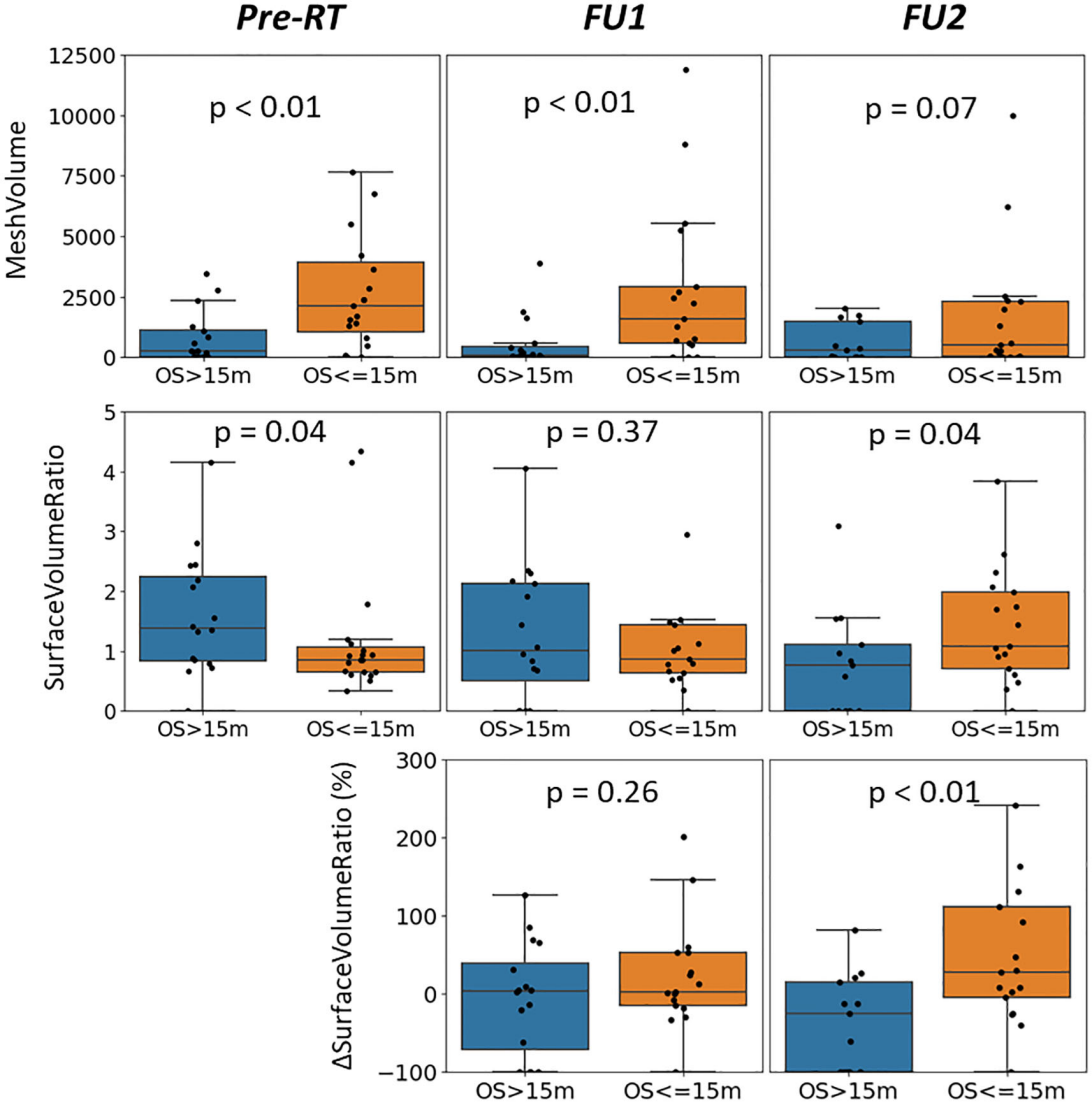
Medians of the relative delta of (upper row) shape and first order radiomics features and (lower row) texture radiomics features (full feature names listed in **Table 3**), separated by longer and shorter survival groups (OS > 15 months or OS <= 15 months), for patients with unmethylated MGMT status.

**Figure 2** displays median values of delta radiomics features found to have a significant difference for patients with unmethylated MGMT status, organized by survival groups. This visualization illustrates the changes over early FU time points. Demonstrating the spread of data points, **Figure 3** provides a detailed representation of individual data points for two of the features, MeshVolume, SurfaceVolumeRatio, and the ΔSurfaceVolumeRatio at pre-RT, FU1, and FU2 time points. Additional plots showcasing other selected radiomics features can be found in the **Supplementary Material** (**Supplementary Figures S2**-**S9**). The Mann-Whitney Test was used to calculate the p-value for each radiomic feature between the two survival groups.

**Figure 4:**
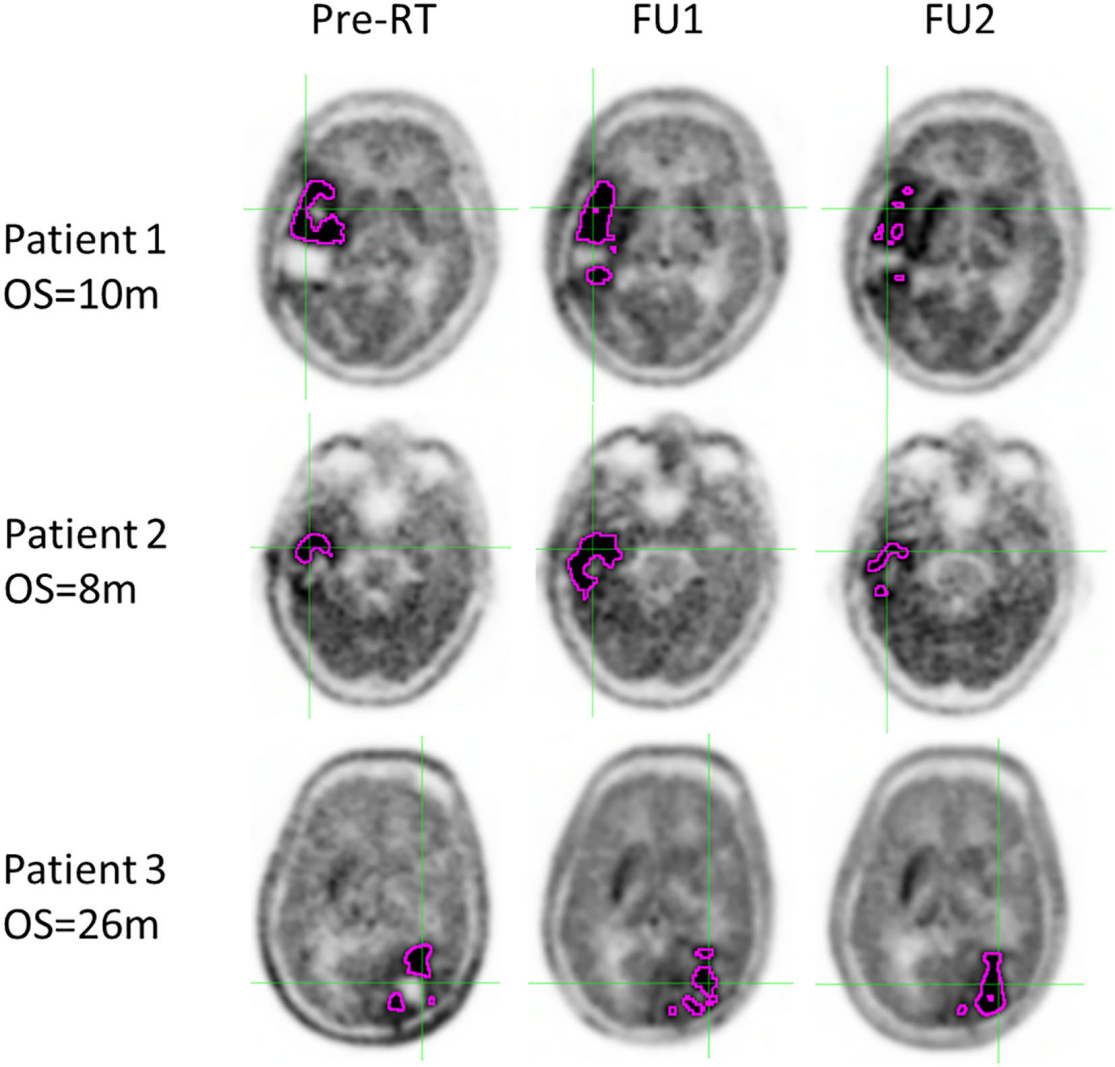
Distribution of MeshVolume (top row), SurfaceVolumeRatio (middle row) and ΔSurfaceVolumeRatio (bottom row) of the patients with unmethylated MGMT status, grouped by survival groups and plotted at different time points (left: pre-RT, middle: FU1, right: FU2), with p-value between longer and shorter survival groups reported.

To visually illustrate tumor changes, co-registered PET images for three example patients are shown in **Figure 4** for time points preRT, FU1, and FU2. Patient 1, who had a short OS, exhibited a small change in tumor volume and SurfaceVolumeRatio at FU1, but the tumor volume decreased and the SurfaceVolumeRatio increased at FU2. Patient 2, who also had a short OS, showed a significant increase in tumor volume after RT and nearly identical SurfaceVolumeRatio at FU1, which then increased at FU2. Patient 3, who had a long OS, demonstrated a decrease in SurfaceVolumeRatio at FU2. For reference, the FU1 timepoint with respect to the last session of RT was consistently at 4 weeks for all three example patients, and the FU2 timepoint with respect to the last session of RT was 2, 2 and 3 months for the patients respectively.

### Survival analysis for RS

Although RS is a dynamic value that updates at each follow-up time point, it exhibits a clear correlation with OS. In the analyses of pre-RT images described in Section 3.2, TBRmax(preRT) was identified as a strong prognostic indicator for OS with a threshold around 3.0. In the RS analysis, TBRmax(preRT) was therefore chosen as the reference baseline. We further assessed the added value of delta features at early FU timepoints. TBRmax(FU) is the TBRmax value measured from FU images. The delta features were evaluated in univariate Cox regression analysis or combined with TBRmax(preRT) in multivariate analysis. While multiple combinations of other delta radiomics features were investigated, none demonstrated a stronger prognostic value than ΔSurfaceVolumeRatio(FU). For clarity and simplicity, **Table 4** summarizes the C-index for various feature combinations at different timepoints, highlights the results for ΔSurfaceVolumeRatio(FU), which gives the best achieved results when combined with TBRmax(preRT).

**Table 4:** Summary of C-index from Cox regression analysis with feature sets at different time points.

Feature set	Features	C-index		
		preRT	FU1	FU2
1	TBRmax (preRT)	0.66	0.68	0.66
2	TBRmax (FU)	N/A	0.68	0.61
3	ΔSurfaceVolumeRatio (FU)	N/A	0.58	0.7
4	TBRmax (preRT), ΔSurfaceVolumeRatio (FU)	N/A	0.68	0.73
5	TBRmax (preRT), TBRmax (FU), ΔSurfaceVolumeRatio (FU)	N/A	0.69	0.73

## Discussion

Glioblastoma, one of the most aggressive tumors, presents significant treatment challenges. Achieving an optimal balance between maximizing tumor control and minimizing toxicity is critical. While dose escalation targeting tumor heterogeneity has shown promising results, not all patients benefit equally. Identifying those who are most likely to benefit from dose escalation is essential for advancing individualized treatment. Additionally, informed post-treatment management plays a vital role in maximizing patient survival. Static medical images offer a momentary snapshot of the tumor’s status. Examining the time series evolution of these images provides a valuable opportunity to investigate how the tumor responds to significant interventions, such as radiation and chemotherapy. Delta features from quantitative imaging serve as a tool specifically designed to discover and quantify these responses over time, capturing imaging characteristics that are often imperceptible to the naked eye. In our study, we extracted quantitative features from ^18^F-DOPA PET images at multiple time points. We identified interpretable pre-RT prognostic features that could further stratify patients with similar pathological biomarkers for DERT. Additionally, we examined significant changes in the quantitative features between pre-RT and post-RT timepoints, evaluating their prognostic value in predicting remaining survival after follow-up.

As consistently observed in various clinical studies, MGMT methylation serves as a robust biomarker correlated with patient survival (2, 5), a trend reaffirmed in this study. MGMT methylation status is a strong prognosis biomarker for the OS in this study, with significantly different median OS for patients with different MGMT methylation status (**Table 1**). However, even with the same MGMT unmethylated status, patients show a wide variation in OS and may respond differently to DERT, as illustrated in **Figures 1**, **2**. It is valuable to further stratify patients using imaging biomarkers in addition to MGMT status. Consistent with previous studies on other amino acid PET tracers (e.g., ^18^FET) (40, 41), TBRmax was identified in this study as a strong pre-irradiation prognostic indicator for OS in patients with unmethylated MGMT. Using a simple threshold of TBRmax = 3.0, these patients can be divided into two distinct survival groups (**Figure 1b**). A slightly more complex model (DT) does not offer superior performance in accuracy for OS grouping. Among patients with high TBRmax, the SS group had a median survival of 13 months, aligning with the median OS of 13.5 months reported in historical cohorts without dose escalation (31), suggesting limited benefit from dose escalation when TBRmax is high. In contrast, the LS group had a significantly longer median survival of 18 months compared to the historical cohort (13.5 months). TBRmax does not show prognostic value to predict OS of the patients with methylated MGMT in our study. The median OS (38 months) of the methylated MGMT subgroup treated with DERT is significantly higher than that of the historical cohort (23.3 months) (31), which may suggest that the patients with methylated MGMT could benefit from dose escalation.

Different survival groups also exhibit different patterns of delta features. We searched for the features showing a significant difference in early FU time points after DERT. **Table 2** reports 8 features identified for the cohort with unmethylated MGMT, while no feature was identified for the cohort with methylated MGMT at the early FU timepoints focused on for this work. Notably, while texture features may reflect certain special characteristics of tumors, they are less intuitive to interpret and can be subject to variation in image quality. For the cohort with unmethylated MGMT, **Figure 2** illustrates the different patterns of response of image features after DERT. Generally, patients with shorter survival exhibit significant change at FU2 time points, not at FU1. Conversely, patients with longer survival demonstrated early responses in certain features, such as tumor volume and energy, with a significant decrease observed at FU1. This indicates that early tumor response to radiation may be a positive prognostic factor. Notably, for features displaying significant changes at FU2, the direction of change differed between the LS and SS groups. For instance, SurfaceVolumeRatio and ZonePercentage decreased at FU2 in the LS group but increased in the SS group. The variation in the timing of the most substantial percentage change suggests the presence of underlying biological mechanisms related to the radiosensitivity of tumors in different patients, highlighting the need for further investigation. As an example, **Figure 3** provides a detailed illustration of the changes in tumor size and SurfaceVolumeRatio, which are among the most interpretable and robust features. SurfaceVolumeRatio has a strong correlation with tumor shape heterogeneity. While the difference of the tumor sizes between LS and SS groups decreased after treatment, the changes of SurfaceVolumeRatio moved in different directions, indicating different responses of the two groups after DERT. The enlarged SurfaceVolumeRatio associated with SS suggested increasing shape heterogeneity, which might be a prognostic indicator of treatment response. Interestingly, this increase in SurfaceVolumeRatio is not observed in patients with methylated MGMT, in either LS or SS groups at FU1 or FU2, although the possibility of such changes at later FU time points was not evaluated in this work. This may indicate differential biologic response to treatment between cohorts. These findings underscore the significance of considering temporal changes in radiomic features and propose potential implications for understanding tumor response and prognosis in the context of treatment interventions. Delta radiomics can also benefit risk analysis for the remaining survival at FU timepoints, as illustrated in **Table 4**. With treatment effects such as radiation included necrosis, the prognostic value of TBRmax may not hold in the FU images. For example, a significant drop of C-index based on TBRmax(FU) is observed at FU2, while including the delta radiomics can increase C-index and boost the accuracy of risk analysis. Among all the identified delta features, ΔSurfaceVolumeRatio at FU2 shows the most prognostic value. The prognostic delta features provide an opportunity to evaluate treatment response and offer the potential to more effectively differentiate true progression from treatment effects. This is particularly important in cases where traditional criteria, often based on the size of the hyperintense region, may be unreliable due to the effects of high-dose radiation therapy. By leveraging these features, clinicians may enable the timely initiation of salvage treatments while avoiding the premature discontinuation of effective therapies or delays in addressing true progression.

In this study, quantitative image analysis was conducted for both unmethylated and methylated MGMT cohorts, revealing distinct phenotypes between the two groups. The features identified as prognostic for unmethylated MGMT (including TBRmax) generally did not demonstrate prognostic value for methylated MGMT. While further statistical validation is needed, these findings suggest that imaging studies may need to be conducted separately based on MGMT status, as image features in PET images appear to evolve differently in cohorts with different MGMT status.

Several limitations should be acknowledged in this study. Firstly, the small sample size represents a primary constraint. Given that ^18^F-DOPA PET imaging is an emerging technique not yet approved by the FDA for glioblastoma, its availability, particularly for FU images, is limited in USA. This imposes limitations on the complexity of radiomics feature selection, modeling, and validation. For mitigation, we chose the simple models based on the most reproducible and interpretable features to avoid overfitting. The reported work would benefit from validation and improvement through additional data, which could be obtained from additional prospective trials utilizing the ^18^F-DOPA PET tracer. Secondly, this study tries to identify the features with the most significant changes before and after RT, instead of identifying all the features which may exhibit difference with statistical significance. With limited statistics, the threshold used to categorize the difference has not been optimized and is subject to further improvement in the future. Thirdly, the focus of this study is on a single cohort of patients treated with DERT, and the applicability of the identified features to other cohorts requires further investigation. Fourthly, the timing of FU2 had a mean of 2.7 months following the completion of RT with a standard deviation of 0.6 months. Although we consider it as an acceptable variability given the constraints in clinical settings and consistency with National Comprehensive Cancer Network (NCCN) recommendations, reduced variability in the FU2 timepoint could be beneficial for quantitative imaging analysis. Finally, the study primarily concentrates on shape and intensity features. While texture features could offer critical and complementary information (as possibly indicated by the exploratory Random Forest model presented in the **Supplementary Material**), they are not extensively explored due to concerns about interpretability and susceptibility to image quality. In the future, with the availability of more data, a revisit and deeper exploration of texture features are warranted.

## Conclusions

This study explores the potential to further stratify glioblastoma patients using radiomics features derived from ^18^F-DOPA PET images and applies delta radiomics features to better understand early treatment responses. Leveraging the high sensitivity of 18F-DOPA PET imaging for glioblastoma, the selected radiomics features, especially TBRmax, from pre-RT images can effectively stratify the patient cohort with un-methylated MGMT and wild-type IDH1, identifying the patients who may benefit most from DERT. The unique characteristics of delta features illuminate distinct treatment response patterns among different survival groups in glioblastoma patients. The observed varied trends in the changes of radiomics feature provide insight into the evolving heterogeneity of tumors following DERT. These findings furnish a valuable tool for evaluating treatment efficacy at an individual patient level and guiding targeted post-treatment interventions when needed. The distinct response pattern also suggests that ^18^F-DOPA PET images have the potential to be utilized for treatment adaptation during treatment courses.

Looking ahead, we will utilize time series data to separate treatment effects from tumor progression, another important and challenging problem in glioblastoma treatment. We will also expand the research with the addition of MR images. Additionally, we also aim to enlarge the cohort size by incorporating data from diverse protocols and institutions, including the newly launched prospective clinical trial (NCT05781321). This expansion will validate the robustness of our models and prognostic features, paving the way for the development of multi-variable models for more personalized approaches to medicine. With various regimens, we will also investigate whether radiation dose and fractionation influence treatment response. We strongly believe that amino-acid PET tracers, including ^18^F-DOPA, hold immense potential in enhancing glioblastoma treatment and management, presenting exciting opportunities for both research and clinical applications.

## Data Availability

The original contributions presented in the study are included in the article/**Supplementary Material**. Further inquiries can be directed to the corresponding authors.
