# Supplementary material for: Analysis of imaging signatures in 18F-DOPA PET of glioblastoma treated with dose-escalated radiotherapy
Source: Front Oncol. 2025 Aug 13;15:1623313. doi: 10.3389/fonc.2025.1623313 (PMC12380700; doi:10.3389/fonc.2025.1623313)
Supplement: Supplementary file 1 [file DataSheet1.pdf]

Supplementary Material

1 Supplementary Figures and Tables

1.1 Supplementary Tables

Supplementary Table S1: The extracted features in this study include 26 shape, 19 first-order and 70 texture features from each scan. And the texture features can be furthered categorized into 24 gray level co-occurrence matrix (GLCM), 16 gray level dependence matrix (GLDM), 16 gray level run length matrix (GLRLM), and 14 gray level size zone matrix (GLSZM)). The detailed definition of the features can be found in https://pyradiomics.readthedocs.io/en/latest/features.html.

Feature Categories	Feature Subcategories	Feature Names
Shape		Elongation, Flatness, LeastAxisLength, MajorAxisLength, Maximum2DDiameterColumn, Maximum2DDiameterRow, Maximum2DDiameterSlice, Maximum3DDiameter, MeshVolume, MinorAxisLength, Sphericity, SurfaceArea, SurfaceVolumeRatio, VoxelVolume
First order		10Percentile, 90Percentile, Energy, Entropy, InterquartileRange, Kurtosis, Maximum, MeanAbsoluteDeviation, Mean, Median, Minimum, Range, RobustMeanAbsoluteDeviation, RootMeanSquared, Skewness, TotalEnergy, Uniformity, Variance
Texture	*gray level co-occurrence matrix (GLCM)*	Autocorrelation, ClusterProminence, ClusterShade, ClusterTendency, Contrast, Correlation, DifferenceAverage, DifferenceEntropy, DifferenceVariance, Id, Idm, Idmn, Idn, Imc1, Imc2, InverseVariance, JointAverage, JointEnergy, JointEntropy, MCC, MaximumProbability, SumAverage, SumEntropy, SumSquares
	gray level dependence matrix (GLDM)	DependenceEntropy, DependenceNonUniformity, DependenceNonUniformityNormalized, DependenceVariance, GrayLevelNonUniformity, GrayLevelVariance, HighGrayLevelEmphasis, LargeDependenceEmphasis, LargeDependenceHighGrayLevelEmphasis, LargeDependenceLowGrayLevelEmphasis, LowGrayLevelEmphasis, SmallDependenceEmphasis, SmallDependenceHighGrayLevelEmphasis

	gray level run length matrix (GLRLM)	SmallDependenceLowGrayLevelEmphasis, GrayLevelNonUniformity, GrayLevelNonUniformityNormalized, GrayLevelVariance, HighGrayLevelRunEmphasis, LongRunEmphasis, LongRunHighGrayLevelEmphasis, LongRunLowGrayLevelEmphasis, LowGrayLevelRunEmphasis, RunEntropy, RunLengthNonUniformity, RunLengthNonUniformityNormalized, RunPercentage, RunVariance, ShortRunEmphasis, ShortRunHighGrayLevelEmphasis, ShortRunLowGrayLevelEmphasis
	gray level size zone matrix (GLSZM))	GrayLevelNonUniformity, GrayLevelNonUniformityNormalized, GrayLevelVariance, HighGrayLevelZoneEmphasis, LargeAreaEmphasis, LargeAreaHighGrayLevelEmphasis, LargeAreaLowGrayLevelEmphasis, LowGrayLevelZoneEmphasis, SizeZoneNonUniformity, SizeZoneNonUniformityNormalized, SmallAreaEmphasis, SmallAreaHighGrayLevelEmphasis, SmallAreaLowGrayLevelEmphasis, ZoneEntropy, ZonePercentage, ZoneVariance

Supplementary Table S2: The list of identified delta features showing significant differences between preRT and postRT images, with a brief description of each feature. The detailed description and mathematical definition of the features can be found in https://pyradiomics.readthedocs.io/en/latest/features.html.

Feature name	Simple Description
Shape_MeshVolume	Total tumor volume calculated with a triangle mesh.
Shape_SurfaceVolumeRatio	Surface divided by volume
FirstOrder_Energy	A measure of the magnitude of voxel values in an image.
GLDM_ GrayLevelNonUniformity	Measures the similarity of gray-level intensity values in the image, where a lower value correlates with greater similarity in intensity.
GLRLM_ ShortRunHighGrayLevelEmphasis	Measures the joint distribution of shorter run lengths.
GLCM_Contrast	Measures the disparity in intensity values among neighboring voxels.

GLSZM_ SmallAreaHighGrayLevelEmphasis	Measures the proportion in the image of the joint distribution of smaller size zones with higher gray-level values.
GLSZM_ ZonePercentage	Measures the coarseness of the texture by taking the ratio of the number of zones, defined as the number of connected voxels that share the same gray level intensity, with the number of voxels.

Supplementary Table S3: Patient Specific FU time and the delta of SurfaceVolumeRatio values, grouped by their survival groups.

**Anonymous**	**FU1 time to Last Fx (days)**	**Delta Surface Volume Ratio (FU1)**	**FU2 time to Last Fx (days)**	**Delta Surface Volume Ratio (FU2)**	**Survival Group**
RT_FDOPA10	27	-7.84	66	131.29	OS<=15m
RT_FDOPA22	34	12.35	79	2.62	OS<=15m
RT_FDOPA23	31	-18.29	66	46.97	OS<=15m
RT_FDOPA34	33	-0.37	68	28.19	OS<=15m
RT_FDOPA35	33	-100.00	82	-39.73	OS<=15m
RT_FDOPA37	29	24.06	71	30.38	OS<=15m
RT_FDOPA42	30	1.65	62	8.03	OS<=15m
RT_FDOPA44	27	-32.84	69	-25.13	OS<=15m
RT_FDOPA45	41	52.67	76	111.99	OS<=15m
RT_FDOPA47	30	53.44	65	309.24	OS<=15m
RT_FDOPA55	23	-29.29	86	-100.00	OS<=15m
RT_FDOPA56	33	145.59	85	241.62	OS<=15m
RT_FDOPA58	34	27.49	97	92.52	OS<=15m
RT_FDOPA68	41	-14.25	87	-26.40	OS<=15m
RT_FDOPA69	39	60.13	67	163.42	OS<=15m
RT_FDOPA70	27	2.93	55	7.99	OS<=15m
RT_FDOPA76	29	200.62	58	-4.78	OS<=15m
RT_FDOPA01	38	126.14	55	81.46	OS>15m
RT_FDOPA11	33	4.30	68	20.47	OS>15m
RT_FDOPA12	38	4.38	94	-24.80	OS>15m
RT_FDOPA19	42	69.51			OS>15m
RT_FDOPA39	29	-100.00	89	-100.00	OS>15m
RT_FDOPA40	37	2.24	104	-12.96	OS>15m
RT_FDOPA41	35	8.84			OS>15m
RT_FDOPA43	29	30.66	61	15.67	OS>15m
RT_FDOPA46	39	-100.00	105	-100.00	OS>15m
RT_FDOPA48	28	-61.92	63	-60.67	OS>15m

RT_FDOPA49	30	65.48	83	-100.00	OS>15m
RT_FDOPA53	30	85.47	97	-100.00	OS>15m
RT_FDOPA59	38	-13.04	87	26.32	OS>15m
RT_FDOPA60	32	-100.00			OS>15m
RT_FDOPA64	32	-20.12	96	-12.88	OS>15m
RT_FDOPA67	31	-100.00	91	-100.00	OS>15m

1.2 Supplementary Figures

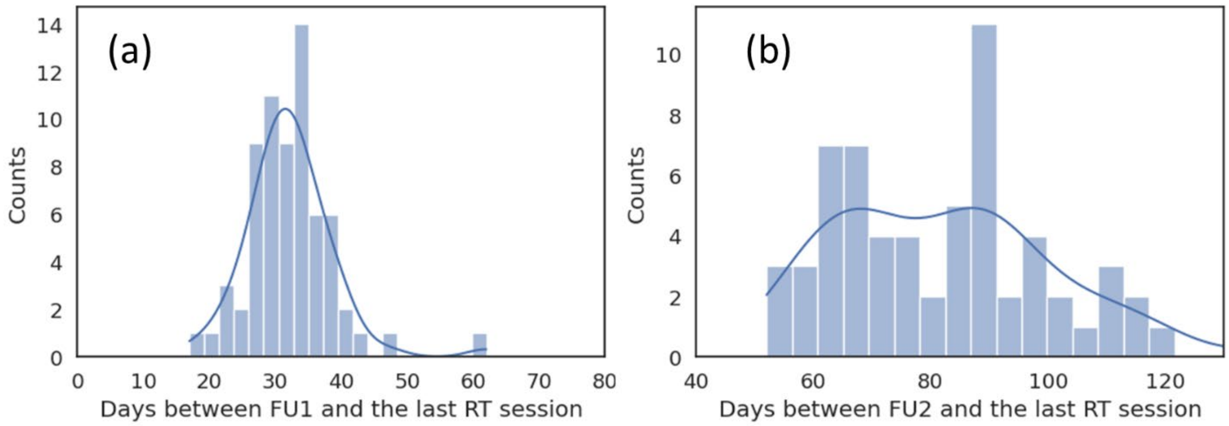

Supplementary Figure S1: Distribution of days between (a) FU1 and (b) FU2 imaging date and the date of the last session of Radiotherapy for the patients in this study.

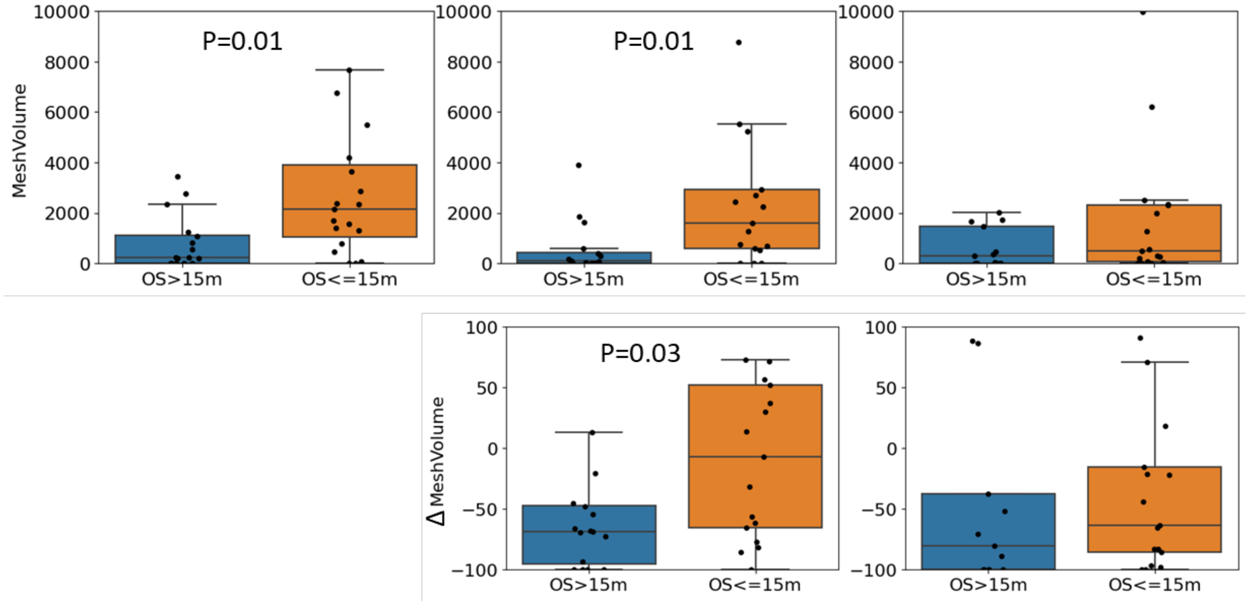

Supplementary Figure S2: (Top) MeshVolume organized by survival groups at preRT (left), FU1 (middle), and FU2 (right); (Bottom) Delta of MeshVolume at FU1 (middle) and FU2 (right), for patients with unmethylated MGMT.

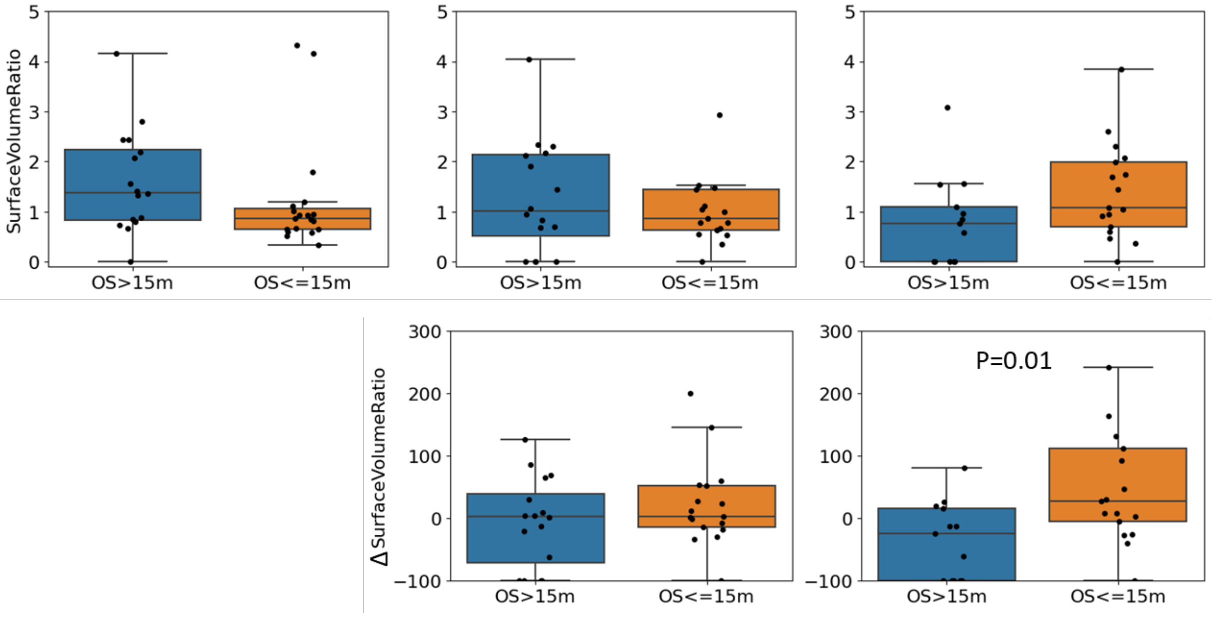

Supplementary Figure S3: (Top) SurfaceVolumeRatio organized by survival groups at preRT (left), FU1 (middle), and FU2 (right); (Bottom) Delta of SurfaceVolumeRatio at FU1 (middle) and FU2 (right), for patients with unmethylated MGMT.

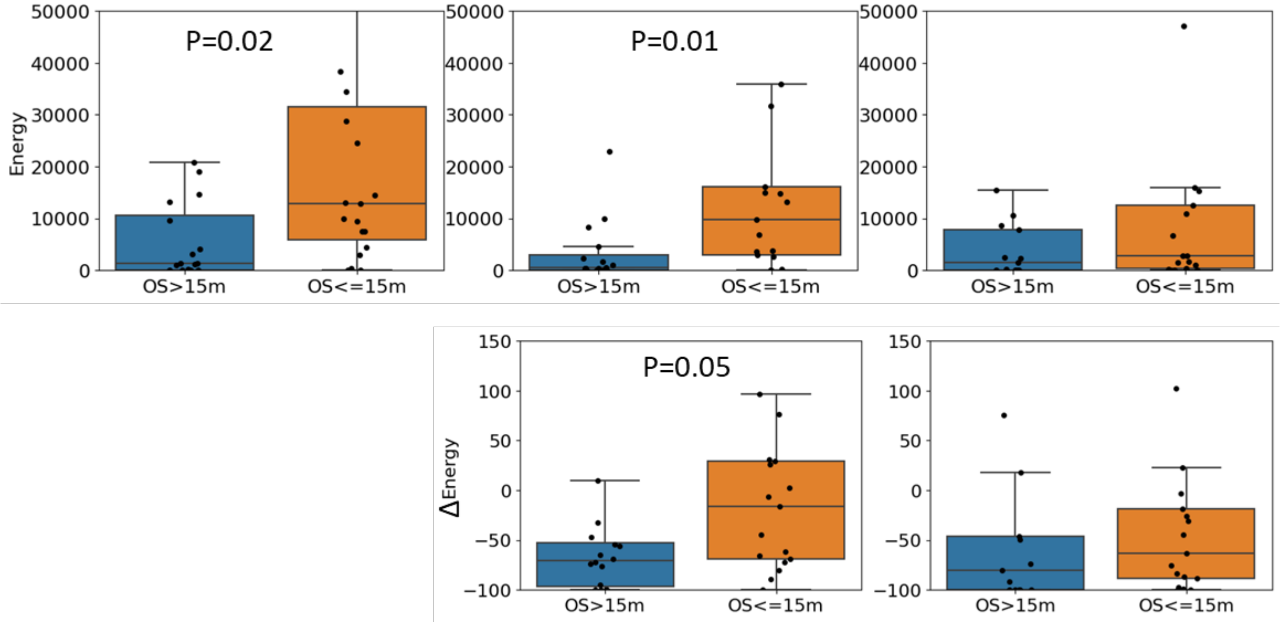

Supplementary Figure S4: (Top) Energy organized by survival groups at preRT (left), FU1 (middle), and FU2 (right); (Bottom) Delta of Energy at FU1 (middle) and FU2 (right), for patients with unmethylated MGMT.

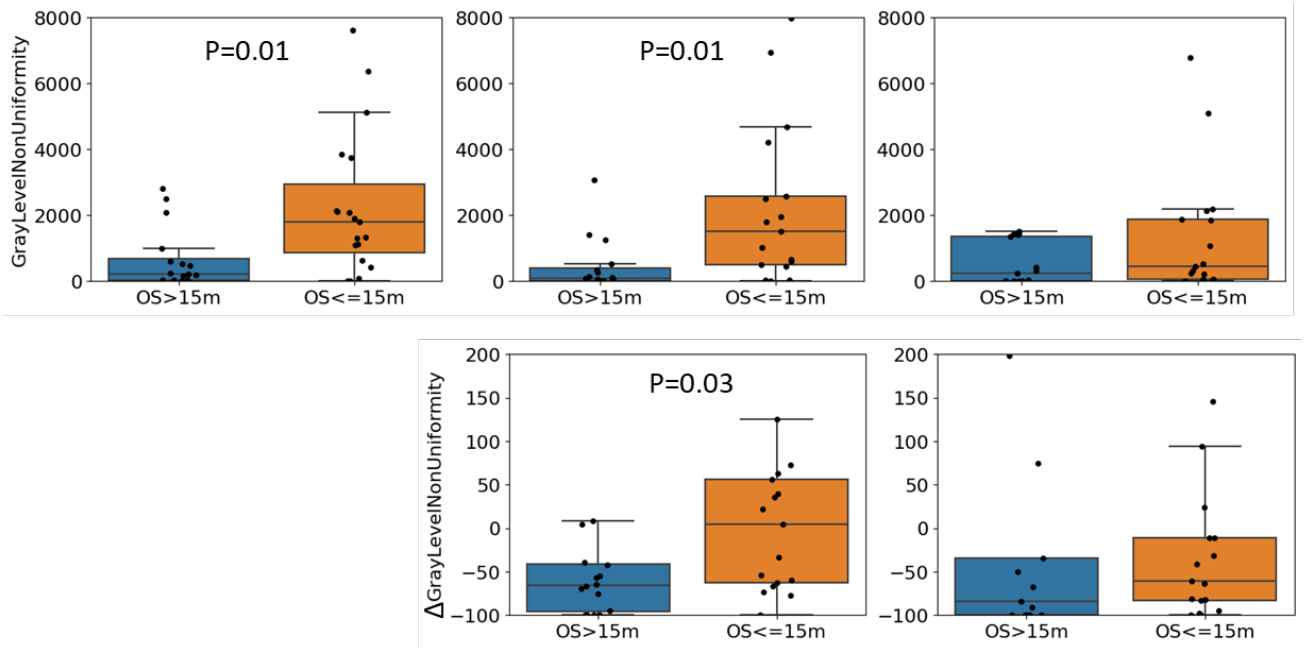

Supplementary Figure S5: (Top) GrayLevelNonUniformity organized by survival groups at preRT (left), FU1 (middle), and FU2 (right); (Bottom) Delta of GrayLevelNonUniformity at FU1 (middle) and FU2 (right), for patients with unmethylated MGMT.

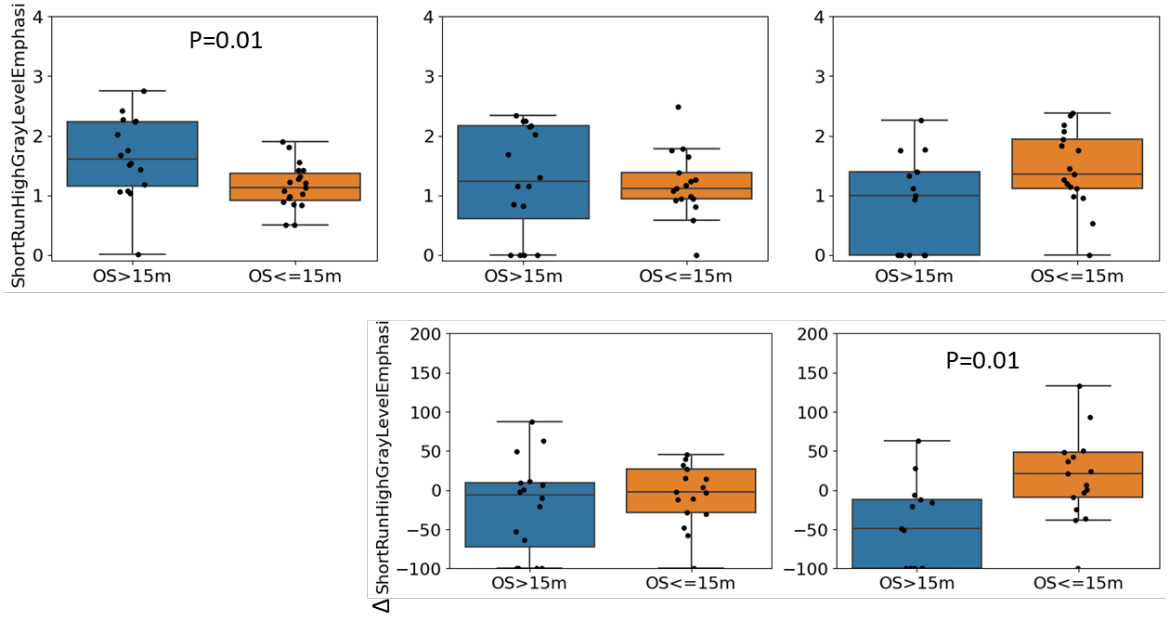

Supplementary Figure S6: (Top) ShortRunHighGrayLevelEmphasis organized by survival groups at preRT (left), FU1 (middle), and FU2 (right); (Bottom) Delta of ShortRunHighGrayLevelEmphasis at FU1 (middle) and FU2 (right), for patients with unmethylated MGMT.

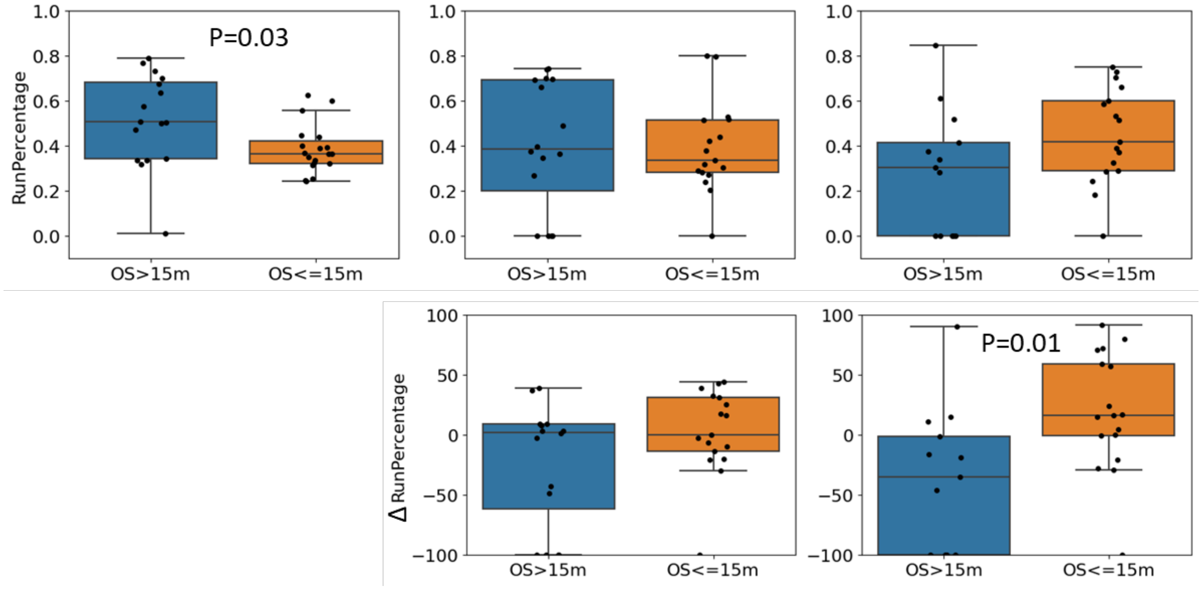

Supplementary Figure S7: (Top) RunPercetage organized by survival groups at preRT (left), FU1 (middle), and FU2 (right); (Bottom) Delta of RunPercetage at FU1 (middle) and FU2 (right), for patients with unmethylated MGMT.

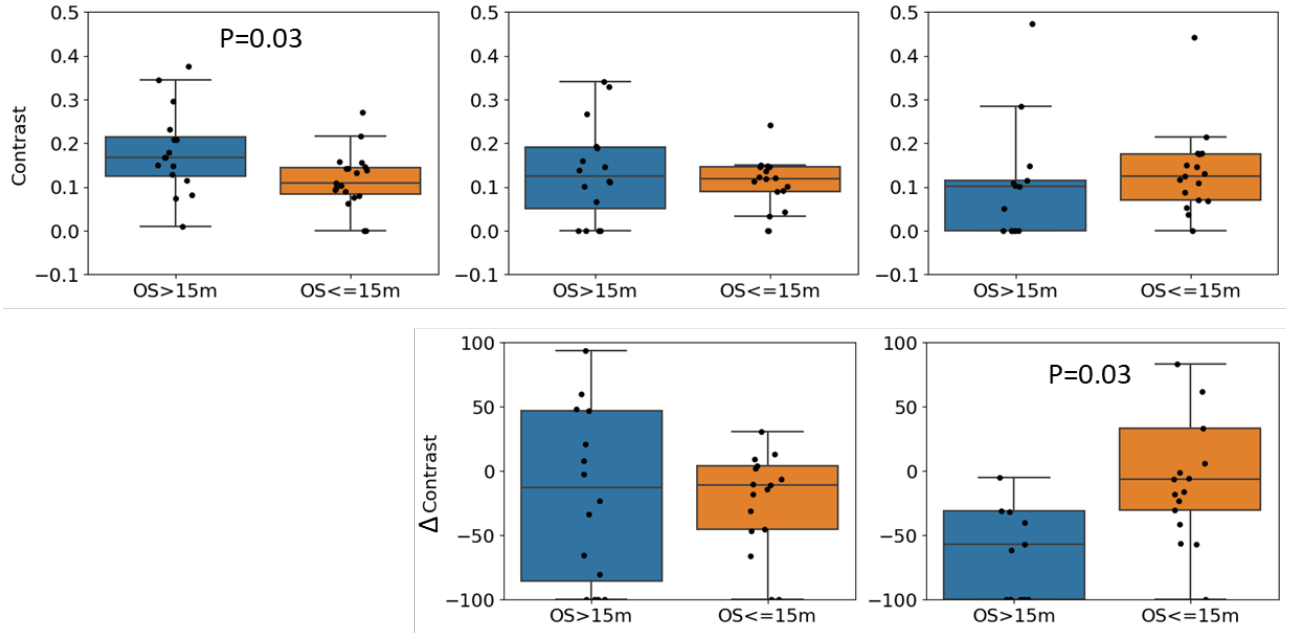

Supplementary Figure S8: (Top) GLCM_Contrast organized by survival groups at preRT (left), FU1 (middle), and FU2 (right); (Bottom) Delta of GLCM_Contrast at FU1 (middle) and FU2 (right), for patients with unmethylated MGMT.

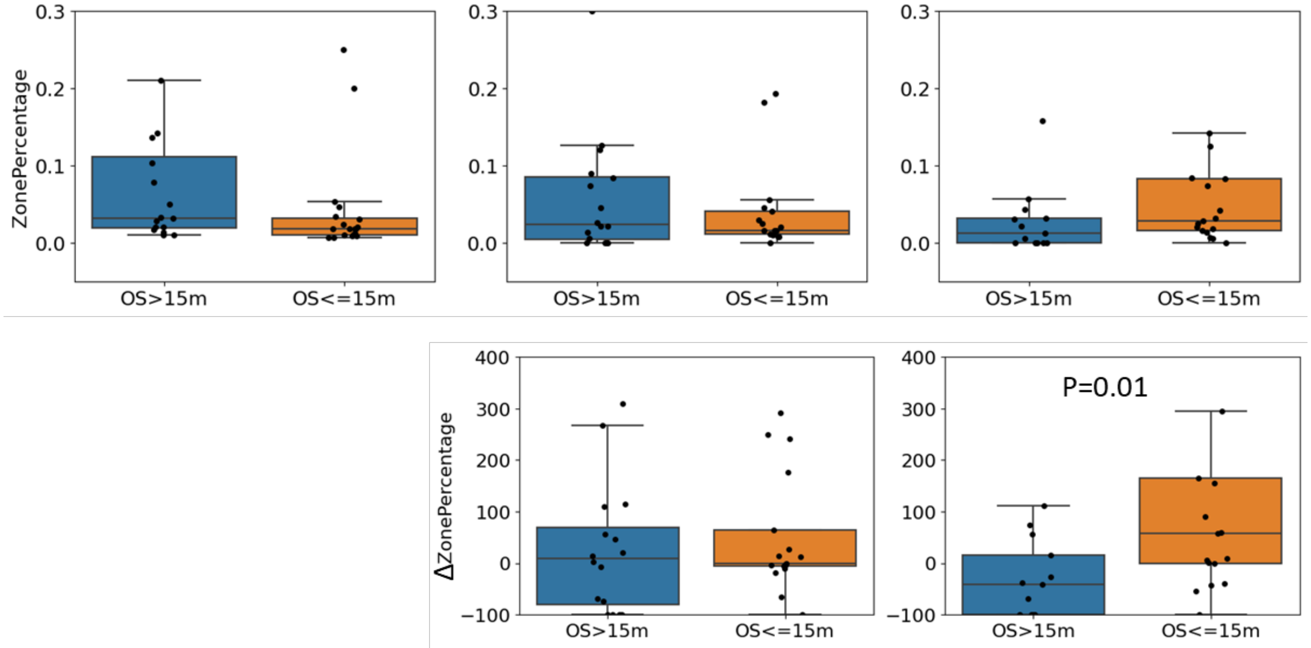

Supplementary Figure S9: (Top) ZonePercentage organized by survival groups at preRT (left), FU1 (middle), and FU2 (right); (Bottom) Delta of ZonePercentage at FU1 (middle) and FU2 (right), for patients with unmethylated MGMT.

1.3 Supplementary Analysis

To explore the discriminative potential of radiomic features for survival stratification, an exploratory Random Forest (RF) classifier was trained using all shape, first-order, and texture features extracted from pre-radiotherapy (pre-RT) ^{18}F-DOPA PET images. The model was developed using the same training cohort in the main text, with 100 trees, a maximum depth of 5, and class-balanced weighting. Feature importance was determined by the mean decrease in impurity across the ensemble.

The analysis highlighted several texture features among the most informative, as shown in Supplementary Table S4. These findings suggest that tumor heterogeneity, as captured by texture metrics on ^{18}F-DOPA PET, may provide relevant information, complementary to the shape and first-order features, for survival group differentiation. But the texture features are often affected by the image quality. Their robustness needs further validation. The interpretability of the texture features may also be more difficult than the interpretability of shape and first-order features.

Supplementary Table S4: The importance of top 10 features selected by Random Forest model.

Feature Categories	Feature Subcategories	Feature Names	Relative Importance
Texture	GLCM	MCC	0.049
Texture	GLRLM	LongRunLowGrayLevelEmphais	0.035
First Order		10Percentile	0.035
Shape		Flatness	0.031
Texture	GLCM	Idm	0.029
Texture	GLRLM	LongRunEmphasis	0.028
Texture	GLCM	Idn	0.026
Texture	GLSZM	SizeZoneNonUniformityNormal	0.026
Texture	GLCM	Contrast	0.024
Texture	GLCM	MaximumProbability	0.024

Evaluation of the RF model on the held-out test cohort demonstrated strong performance, with an accuracy of 0.89 and an ROC AUC of 0.90. The ROC curve on the held-out test cohort is shown in Supplementary Figure S10.

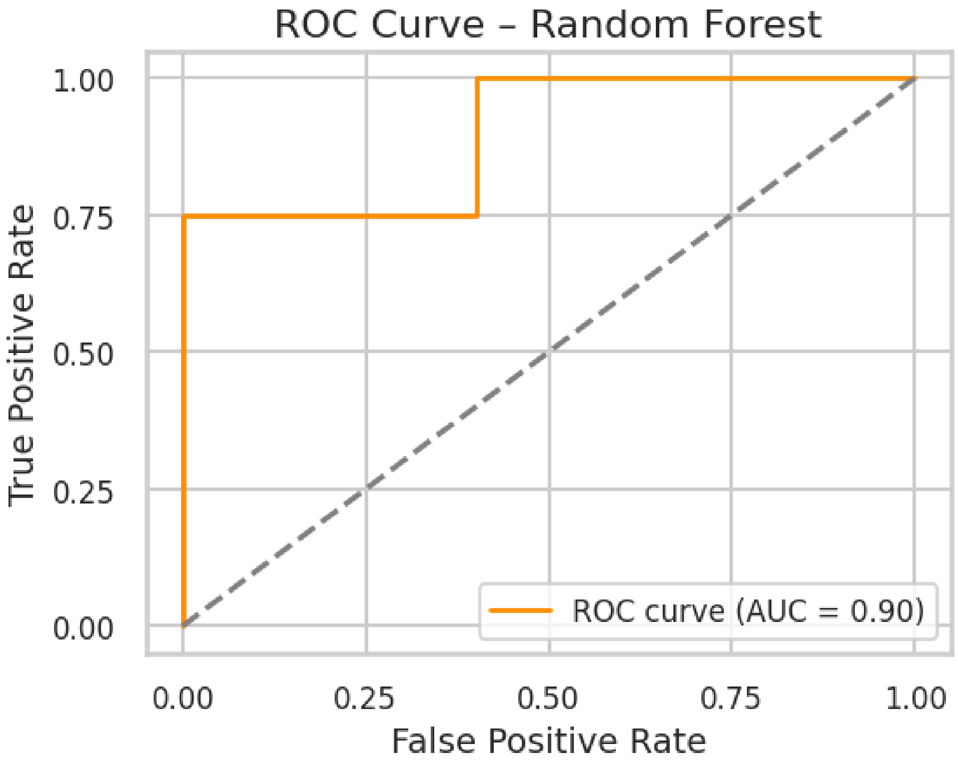

Supplementary Figure S10: ROC curve of the exploratory Random Forest model evaluated on the held-out test cohort for survival group classification using radiomic features, including texture features, extracted from pre-treatment ^{18}F-DOPA PET.
